# Prognostic significance of fascin expression in advanced colorectal cancer: an immunohistochemical study of colorectal adenomas and adenocarcinomas

**DOI:** 10.1186/1471-2407-6-241

**Published:** 2006-10-09

**Authors:** Yosuke Hashimoto, Marek Skacel, Ian C Lavery, Abir L Mukherjee, Graham Casey, Josephine C Adams

**Affiliations:** 1 Department of Cell Biology, Lerner Research Institute, The Cleveland Clinic, 9500 Euclid Avenue, Cleveland, OH 44195, USA; 2 Department of Anatomic Pathology, The Cleveland Clinic, 9500 Euclid Avenue, Cleveland, OH 44195, USA; 3 Department of Colorectal Surgery, The Cleveland Clinic, 9500 Euclid Avenue, Cleveland, OH 44195, USA; 4 Department of Cancer Biology, Lerner Research Institute, The Cleveland Clinic, 9500 Euclid Avenue, Cleveland, OH 44195, USA; 5 Department of Molecular Medicine, Cleveland Clinic Lerner College of Medicine at Case Western Reserve University, The Cleveland Clinic, 9500 Euclid Avenue, Cleveland, OH 44195, USA

## Abstract

**Background:**

Fascin is an actin bundling protein with roles in the formation of cell protrusions and motility of mesenchymal and neuronal cells. Fascin is normally low or absent from epithelia, but is upregulated in several epithelial neoplasms where it may contribute to an invasive phenotype. Here, we report on the prevalence and potential clinical significance of fascin expression in relation to the progression of colorectal adenocarcinoma and to tumor cell proliferation as measured by Ki67 index.

**Methods:**

Conventional tissue sections of 107 colorectal adenomas and 35 adenocarcinomas were analyzed by immunohistochemistry for fascin and Ki67 expression.

Fascin expression and Ki67 proliferation index were also investigated by use of a tissue microarray containing cores from a further 158 colorectal adenocarcinomas and 15 adenomas linked to a CCF, IRB-approved database with a mean of 38 months of clinical follow-up. Survival analysis was carried out by the Kaplan-Meier and Cox regression methods.

**Results:**

Fascin was not expressed by the normal colonic epithelium. In conventional sections, 16% of adenomas and 26% of adenocarcinomas showed fascin expression in greater than 10% of the tumor cells. In the clinically-annotated tumors, fascin immunoreactivity was more common in tumors located in the proximal colon (p = 0.009), but was not associated with age, gender, or TNM stage. Patients with stage III/IV adenocarcinomas (n = 62) with strong fascin immunoreactivity had a worse prognosis than patients with low or absent fascin, (3-year overall survival of 11% versus 43% for fascin-negative patients; p = 0.023). In adenomas, fascin and Ki67 tended to be inversely correlated at the cellular level; this trend was less apparent in adenocarcinomas.

**Conclusion:**

Fascin is upregulated in a proportion of adenomas, where its expression is often focal. Strong and diffuse expression was seen in a subset of advanced colorectal adenocarcinomas that correlated with shorter survival in stage III and IV patients. Fascin may have prognostic value as an early biomarker for more aggressive colorectal adenocarcinomas.

## Background

Colorectal carcinoma (CRC) is the third most lethal malignancy in the United States for both women and men, with an overall 5-year survival rate of around 60% [1]. 106,680 cases of colon and 41,930 cases of rectal cancer are expected to occur in 2006. It is estimated that 55,170 deaths from CRC will occur in 2006, accounting for 10% of all cancer deaths [2]. At present, the only curative treatment is surgical resection: however, it is often impossible to remove all cancer cells, especially those that have invaded the surrounding tissues. The penetration of tumor cells into lymphoid vessels and blood vessels leads to tumor metastasis and ultimately the tumor becomes fatal [3]. The current major method for assessing the risk of metastatic recurrence and need for adjuvant chemotherapy is to examine tumor resection specimens for evidence of metastasis to local lymph nodes. However, this approach may be of limited prognostic value as a sizeable fraction of colorectal carcinomas have innate resistance to chemotherapy and 25% to 30% of the patients presenting with lymph-node negative tumors also develop fatal disease [4]. Therefore, there is an urgent need for more accurate and informative methods of individual risk assessment for patients with CRC, some of which might be based on the molecular properties of the primary tumor itself [5].

Tumor invasion and metastasis are the result of highly coordinated processes that involve multiple intracellular and extracellular factors [6-8]. In part, carcinoma cell migration is enabled by the altered differentiation status of the epithelial cells that includes changes in cell-cell and cell-matrix adhesion properties and in the organization of the actin cytoskeleton [9-12]. With regard to the composition of the cytoskeleton of carcinoma cells, the actin-bundling protein, fascin, has become of great interest due to its functional involvement in cell adhesion and motility [13,14]. Fascin is expressed in mature dendritic cells, mesenchymal cells, endothelial cells and neurons during development and in the adult [15,16]. It is absent from most normal epithelia, but is expressed in multiple epithelial neoplasms, including carcinomas of the pancreas, lung, esophagus, stomach and breast [17-24]. Most strikingly, fascin expression has been associated with a poorer prognosis in carcinomas of the lung, esophagus, stomach and breast [19-21,23]. In node-negative, invasive hereditary breast carcinomas, fascin is frequently expressed by BRCA1-associated tumors [24]. Fascin has also been identified as a component of a gene signature that correlates clinically with breast cancer metastasis to the lung [25].

In cell culture, expression of recombinant fascin in fascin-negative colonic adenocarcinoma cells correlated with increased proliferation, altered beta1 integrin distribution, increased invasive capacity and altered differentiation status [26]. Similar findings have been obtained in other epithelial cells, suggesting that fascin may contribute to a more aggressive tumor phenotype by facilitating carcinoma cell migration and invasion [20,27]. However, with regard to colorectal cancer, an initial study of tumor specimens examined only 10 cases without regard to tumor stage or clinical annotation [26]. Thus, the clinical relevance of fascin expression in CRC remains unclear and it is also unknown whether fascin plays any role in the early development of colorectal carcinoma.

Studies of fascin in multiple cell types have established that its actin-binding properties are regulated by extracellular cues acting both through adhesion receptors and receptor tyrosine kinases [14,16,28-30]. Furthermore, several studies have indicated that fascin expression may be related to the proliferative status of carcinomas. In mouse xenografts, cells from fascin-positive human ovarian carcinomas were more tumorigenic than fascin-negative lines [31]. Colonic epithelial cells engineered to over-express fascin proliferated faster in culture than control cells [26]. Despite these findings, the relationship between fascin expression and cell proliferation in human cancers is currently unclear. In non-small cell lung carcinoma, highly fascin-positive tumors tended to be highly proliferative, as established by Ki67 antibody staining. However, it was also noted that individual Ki67-positive cells stained less strongly for fascin than surrounding tumor cells [19]. In gastric carcinoma, the reverse trend was observed, with a higher Ki67 index in fascin-positive areas compared to fascin-negative areas [21].

Here, we have examined the clinicopathological significance of fascin and Ki67, singly and in combination, in a series of colorectal tumor specimens. By examining whole paraffin-embedded sections, we closely reviewed the localization and topographic relationship of fascin and Ki67 in colonic adenomas and colorectal adenocarcinomas. The potential prognostic significance of fascin expression was assessed by using clinically-annotated samples in a CRC tissue microarray of 158 colorectal adenocarcinomas and 15 adenomas. We report that fascin and Ki67 are most frequently inversely correlated at the cellular level. Fascin is upregulated at the adenoma stage and is of potential prognostic significance as a marker of aggressive colorectal adenocarcinomas.

## Methods

### Patients and surgical specimens: conventional sections

We studied 142 tumor samples that included 107 adenomas (89 sporadic and 18 from patients with familial adenomatous polyposis (FAP)) and 35 adenocarcinomas from an unselected series of cases seen at the Cleveland Clinic between 2004 and 2006. The adenoma samples were derived from 76 patients, comprising 65 patients with sporadic adenomas and 11 FAP patients. Adenomas with high grade dysplasia were not included in the set and only moderately- or poorly-differentiated adenocarcinomas were collected. 76 of the sporadic adenomas were < 2 cm in diameter and 13 were > 2 cm. 16 of the FAP were <2 cm and 2 were > 2 cm in diameter. One representative section of each specimen was selected for study. In adenocarcinomas, this represented the portion of deepest extent. In adenomas, the sections were selected to demonstrate the entire lesion including the head and stalk of the adenoma, where available.

### Tissue microarrays

A custom built instrument equipped with stainless steel thin-wall needles (Beecham Instruments, Hackensack, NJ), was used to take core tissue biopsies from carefully selected, morphologically representative areas of the original paraffin blocks ("donor" blocks) and arrayed into a new "recipient" paraffin block. The precision digital device that equips this instrument allows for precise placement and spacing of the tissue cores into the recipient block. The resulting tissue microarray (TMA) contained a total of 374 cores, from 14 normal colonic epithelia, 15 adenomas and 158 colorectal adenocarcinomas that were diagnosed at The Cleveland Clinic between 1993 and 1999. The majority of the adenocarcinomas were moderately-differentiated and six were poorly-differentiated. Tumors were classified according to standard TNM staging guidelines [32], and the location of tumors was divided into two groups, proximal (cecum, ascending colon, hepatic flexure and transverse colon) and distal (splenic flexure, descending colon, sigmoid colon and rectum). Of the 158 adenocarcinomas, 131 samples were available for scoring after fascin staining; this study group derived from 79 males and 52 females. The median age was 64.5 years (range 32–95 years). Of the 62 patients with stage III or stage IV tumors in the dataset, 19 received no adjuvant treatment, 27 received adjuvant chemotherapy and 16 received combined adjuvant chemotherapy and radiotherapy. To minimize sampling errors, two separate large diameter (1.5 mm) tissue cores of each adenocarcinoma were included in the array, totaling a surface area of 3.5 mm^2 ^per case. Each separate tissue core was assigned a unique TMA location number which was subsequently linked to a CCF Institutional Review Board-approved (IRB-5085) database containing a mean 38 months of clinical follow-up.

### Immunohistochemical staining

Both the conventional paraffin sections and the TMAs were treated similarly. Immunohistochemistry was carried out using a fully automated Ventana Benchmark system (Ventana, Tucson, AZ). Briefly, 4 μm thick unstained sections were placed onto a electrostatically charged glass slide and baked to allow for tissue adherence. The glass slides were pretreated with the recommended pretreatment solution provided by Ventana for tissue deparaffinization and antigen retrieval. After primary antibody incubation and a secondary biotinylated antibody/streptavidin amplification step, antigen detection was carried out by peroxidase/3,3'-diaminobenzidine development. Primary antibodies used in this study were directed against fascin (DAKO, clone 55k-2, dilution 1:50) and Ki67 (Novacastra, clone MM1, dilution 1:5).

### Evaluation of immunohistochemical stainings and scoring

A section without the primary antibody was used as a negative control in each case. A normal tonsil tissue was used as a positive control to confirm fascin immunoreactivity in tonsillar dendritic cells in each series of experiments. Fascin immunoreactivity in each specimen was verified by the staining of endothelial cells in microvessels. Fascin-positive adenoma or adenocarcinoma samples were defined as those showing a cytoplasmic pattern of expression. For the conventional sections, each section was recorded on the basis of the area of fascin staining and was scored on a four point scale: 1), negative; 2), 1% to 10% positive; 3), 10% to 50% positive, and 4), more than 50% positive. For the TMA, this standard scoring scheme was used with minor modifications. Each separate section or tissue core was recorded on basis of the area and intensity of the staining and scored on a 0 to 3+ scale, (0, no staining; 1+, less than 10% of cells moderately to strongly stained, or a weak reactivity in any % of cells; 2+, 10% to 50% of cells moderately or strongly stained; 3+, more than 50% of cells with moderate or strong staining), and the results entered into the research database. In all samples, Ki67 index was scored according to the percentage of cells with positive nuclear staining and was divided into three groups: 1+ (less than 10% of cells with positive nuclei); 2+ (10% to 50% of cells with positive nuclei), and 3+ (more than 50% of cells with positive nuclei).

All slides were evaluated independently by two investigators (YH and MS) without any prior knowledge of the clinical information. When the opinions of the two evaluators differed, a consensus agreement was reached by re-review of the slides and thorough discussion. Where scores differed between two cores on the TMA, the average score was taken.

### Statistical analysis

The immunohistochemical staining scores for fascin and Ki67 in relation to clinicopathological factors were analyzed using chi-squared. The overall survival was defined as that from the date of the operation to the date of death due to cancer. The Kaplan-Meier method was used to determine the probability of survival and the data were analyzed by the log-rank test. Multivariate analysis was performed using the Cox regression model to study the effect of different variables on overall survival. The software StatView for Windows version 5 (SAS Institute, Cary, NC) was used for the analysis. A *p *value of < 0.05 was considered significant.

## Results

### Fascin upregulation is an early event detectable in adenomas

**Figure 1:**
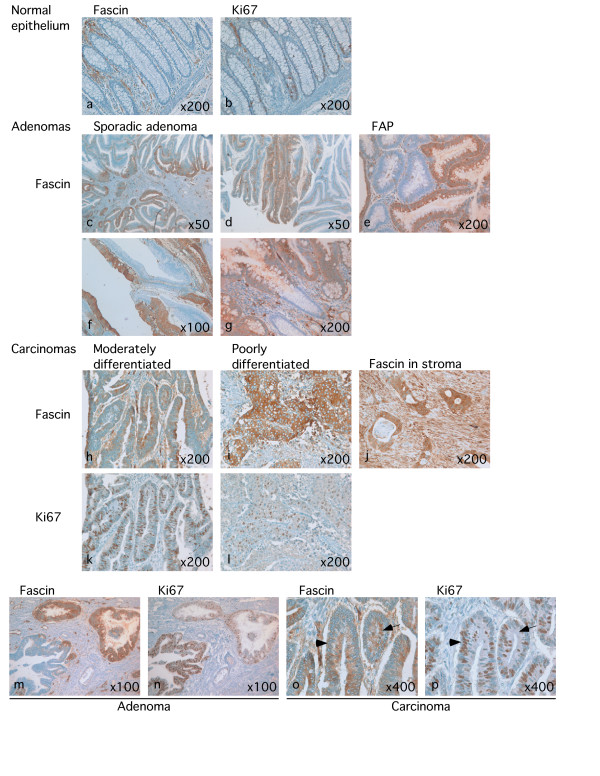
**Expression of fascin and Ki67 in normal colonic epithelium, adenomas and adenocarcinomas**. a, b: immunohistochemical staining for fascin and Ki67 in normal colonic epithelium. c-g: fascin staining in sporadic adenoma (c, d, f, g) and FAP (e). h-l: fascin and Ki67 staining in a moderately-differentiated adenocarcinoma, (h, k), and a poorly-differentiated adenocarcinoma, (i, l). j: stromal staining of fascin in a colorectal adenocarcinoma. m-p: representative examples of fascin, (m, o), and Ki67 staining, (n, p), in continuous sections from a sporadic adenoma, (m, n), and a moderately-differentiated adenocarcinoma, (o, p). Arrows in o and p indicates examples of areas where fascin and Ki67 are inversely correlated; arrowheads indicate areas where staining is over-lapping.

As previously reported, fascin was not expressed in the normal epithelium. Dendritic cells in lymphoid tissue and vascular endothelial cells were positive for fascin and served as internal positive controls (Fig. 1a). To establish whether fascin is upregulated in adenomas, staining for fascin was carried out on whole sections from 107 adenoma specimens, representing both FAP and sporadic cases (Table 1). Of the 89 sporadic adenomas, 19 were fascin-negative. Fifty-four adenomas stained for fascin over less than 10% of the cells, 12 contained between 10% to 50% of fascin-positive cells, and 4 adenomas contained over 50% fascin-positive cells (example in Fig. 1c) (Table 1). Of the 18 FAP samples, fascin staining was detected in less than 10% of the cells in 17 cases and in 10% to 50% of the cells in one case (Fig. 1e). All the sections that included both the head and stalk of the adenoma showed that fascin was expressed most intensely at the base of the crypts, close to the central, stromal stalk region (Fig. 1c). Fascin-positive groups of cells were also present on the lateral walls of many crypts. From sections that included both the central stalk and the edge of the adenoma, these lateral fascin-positive cells were always found to be accompanied by basal fascin-positive cells adjacent to the stalk region (Fig. 1d, example shows a edge region of the same polyp as shown in 1c). Horizontal sections taken through the heads of adenomas demonstrated that the fascin-positive areas were distributed across the whole adenoma (data not shown). In some cases, from both the FAP and sporadic adenomas, entire crypts were positive, yet we also found examples of adenomatous glands that were partly fascin-positive and partly fascin-negative (Fig. 1f). In sections that captured adenomatous structures adjacent to the normal crypt epithelium, a distinct boundary between the fascin-positive adenomatous area and the fascin-negative, normal epithelium was seen (Fig. 1g).

Of 35 colorectal adenocarcinomas examined as conventional sections, 26 showed staining for fascin. In all cases, the staining was cytoplasmic and in most cases the staining intensity was somewhat weaker than that of vascular endothelial cells. The extent of fascin staining in these adenocarcinomas was variable. Many cases showed a patchy distribution of fascin (example in 1 h), whereas in others over than 50% of the cells stained strongly (example in 1i). Overall, 26% (9 out of 35) of the adenocarcinomas contained more than 10% fascin-positive cells (Table 1).

In some tumor specimens we noticed elevated fascin staining in the stroma (Fig. 1j). This staining was not always uniform: in some cases, only some regions of the stroma were positive. This stromal staining appeared independent of tumor staining, because examples of fascin-positive stroma were identified in the presence or absence of fascin staining of the tumor cells (data not shown).

### Fascin expression correlates with a poor prognosis in stage III and IV colon adenocarcinoma patients

**Figure 2:**
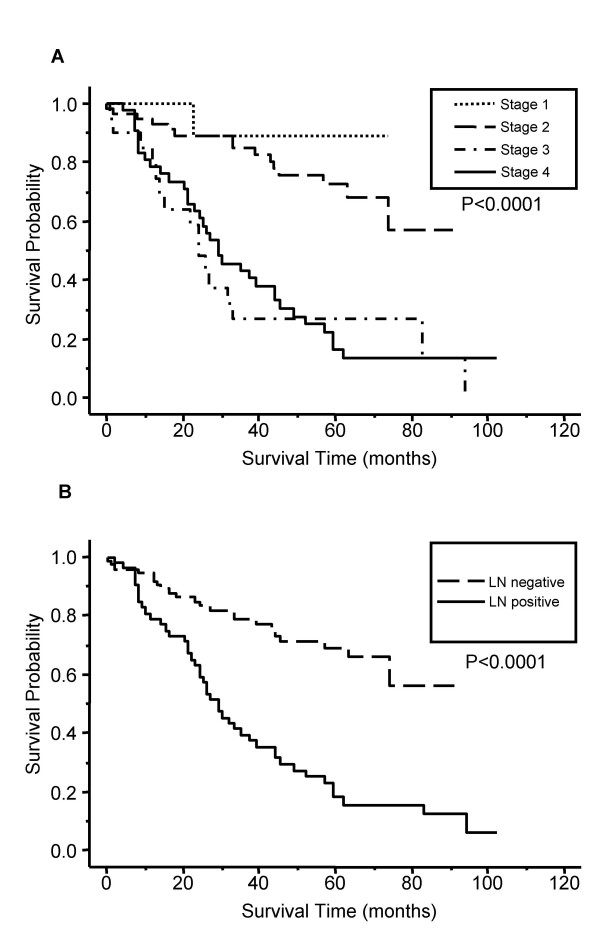
**Kaplan-Meier survival curves of the TMA study population**. The traditional clinicopathological prognostic factors for colorectal carcinoma, TNM stage, (A), and lymph node metastasis, (B), correlate with survival. Both significant at p < 0.0001.

To determine whether fascin expression correlated with the clinical course of CRC, we utilized a TMA of colorectal adenocarcinomas for which clinical annotation was available through an IRB-approved database. The clinical and pathological characteristics of the study group represented the traditional prognostic variables of colorectal adenocarcinomas (Table 2). Tumor stages and lymph node involvement as represented in the study group correlated well with patient survival (Fig. 2). After staining the 158 adenocarcinomas on the array for fascin, 131 adenocarcinoma samples were available for scoring: several cores did not contain proper tissues or were detached during the staining procedure. Within the set of 131 cases, the prognostic variables were represented as in the original dataset (Table 2). Of these 131 specimens, 93 tumors (71%) were negative for fascin and 38 (29%) were positive (Table 3). Elevated stromal fascin was detected in 62 cases. Tumor fascin immunoreactivity was seen most commonly in adenocarcinomas from the proximal colon (p = 0.009), but was not associated with age, gender or TNM stage (Table 3). Elevated stromal fascin staining correlated positively with male gender (p = 0.045) and was inversely correlated with stage (p = 0.01), increasing age (p = 0.035) and lymph node metastasis (p = 0.03).

**Figure 3:**
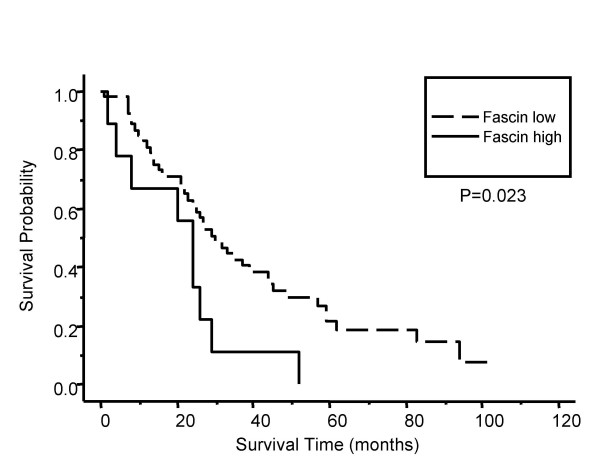
**High fascin expression correlates with a clinically aggressive tumor phenotype**. The Kaplan-Meier survival curves show that stage III and IV patients with highly fascin-positive adenocarcinomas had decreased survival compared to patients with fascin-low or -negative tumors (significant at p = 0.023).

To examine whether tumor fascin expression correlated with patient survival, the fascin-positive cases represented on the TMA were divided into two groups: fascin low, (0 or 1+, n = 113), or fascin high, (2+ or 3+, n = 18). Whereas in the complete set of 131 patients there was no significant difference in survival between patients with low or high fascin expression using the Kaplan-Meier method, (p = 0.226, data not shown), in the 62 patients with stage III or stage IV tumors the survival of patients with tumors that stained strongly for fascin was significantly worse than that of patients with tumors that stained weakly or were negative for fascin (p = 0.023) (Fig. 3). This correlation was independent of whether the patients had received adjuvant treatments or not: patients that had received adjuvant therapies did not have significantly longer survival times than other patients diagnosed with stage III or IV tumors and the fascin-high group of patients included patients both with and without adjuvant therapy (data not shown). Elevated fascin staining in stromal cells did not correlate significantly with patient survival (data not shown).

Cox multivariant analysis demonstrated that lymph node metastasis (N1, 2) was an independent adverse prognostic factor; however, fascin (high), age (>65 years), gender (male) and tumor location (proximal) were not independent prognostic factors in the total group of 131 patients with stage I-IV colorectal adenocarcinoma (Table 4). For the stage III and IV patients, tumor fascin appeared to be an independent adverse prognostic factor (p = 0.039) (Table 4). For stage III and IV patients, all other variables, (age, gender, lymph node metastasis and proximal tumor location), were not independent prognostic factors (Table 4).

### Relationship between fascin and Ki67 staining

We evaluated the possible relationship between fascin and Ki67 staining in normal tissue, adenomas, adenocarcinoma sections and the TMA. In all samples, Ki67 staining was restricted to the nucleus. In the normal tissue, Ki67-positive cells were most apparent in the epithelium, concentrated around the base of the crypt. Staining was only rarely detected in stromal cells (Fig. 1b).

Out of the 131 adenocarcinomas in the TMA, all showed some level of Ki67 staining (Table 5). A detailed comparison of fascin and Ki67 staining at cellular level was made using consecutive sections from 51 adenomas and 12 adenocarcinomas. In the adenomatous crypts, Ki67 expression tended to be highest at the top of each crypt, relatively low in the central region and intermediate at the base (data not shown). Using adenoma cases that stained positively for both fascin and Ki67, we found that the two markers tended to be inversely correlated. Thus, groups of cells with a high Ki67 index were negative for fascin and *vice versa *(Figure 1m, 1n).

In adenocarcinomas, Ki67 staining was more homogenous, with a large fraction of positive tumor cells. The tumor areas always contained many more Ki67-positive cells than the stroma (Fig. 1k, 1l). Close examination of adenocarcinoma specimens that stained positively for both fascin and Ki67 revealed that the two markers were inversely correlated in some areas (example arrowed in Fig. 1o and 1p, which show high magnification fields from the adenocarcinoma in Fig. 1h and 1k), yet appeared to be co-expressed in other areas, even within the same glandular structure (example indicated by arrowhead in Fig. 1o and 1p). Elevated fascin staining in stromal cells did not correlate with Ki67-positive cells (data not shown).

Ki67 staining did not correlate with gender, lymph node metastasis or tumor location, but, as expected, a high Ki67 index correlated with TNM stage (Table 5, p = 0.048). Patients with a high Ki67 index tended to have a poorer prognosis, however this was not statistically significant (p = 0.063) (data not shown). No significant clinical correlation was discovered by correlating Ki67 status with fascin positivity or negativity, either in the whole group of patients or in the stage III and IV patients.

## Discussion

Our study provides several novel insights into the clinical relevance of fascin in colorectal adenocarcinoma. Most significantly, fascin was strongly expressed in similar proportions of adenomas and adenocarcinomas, (16% versus 17% to 26%, respectively), and fascin expression in invasive stage III and IV adenocarcinomas correlated significantly with decreased survival. Given the known roles of fascin in cytoskeletal organization and cell migration, these findings indicate a potential clinical significance of fascin as a marker or prospective therapeutic target for the most aggressive forms of colorectal adenocarcinoma. Our study also clarifies that fascin protein is upregulated in precancerous lesions, of both inherited and sporadic origin. Adenomas are known to be of polyclonal origin [33,34], and additional experimental research will be needed to establish whether and how fascin-staining adenomas give rise to strongly fascin-staining carcinomas. If the two groups are indeed related, fascin could have value as a novel prognostic marker, for example to identify those individuals who should receive additional monitoring or treatment after the detection and surgical removal of an adenoma or invasive adenocarcinoma.

A second novel finding of our study was the highly significant correlation of strong fascin expression with tumor location in the proximal colon (Table 3). The proximal and distal colon have different embryonic origins, distinct blood supplies and innervation [35,36]. Much evidence supports the concept that tumors of the proximal colon have specific clinical and pathological characteristics and arise through distinct genetic and molecular processes [35-37]. Eighty-seven percent of sporadic tumors with microsatellite instability (MIN or MSI tumors) occur in the proximal colon, whereas tumors with chromosomal instability (CIN tumors) predominate in the distal colon [36,37]. It has been suggested that methylating carcinogens are responsible for the origin of MIN tumors in the proximal colon [38]. In general, MIN tumors have a better prognosis [39]. Differing responses of CIN or MIN tumors to chemotherapy have been reported [39,40]. However, in our dataset, those patients with strongly fascin-positive advanced tumors had poorer survival (Figure 3). From Kaplan-Meier analyses we found that fascin-high, proximal tumors did not correlate with reduced survival (unpublished observation). Fascin-high distal tumors were not represented in large enough numbers in the current dataset to obtain a statistically meaningful comparison for this group alone. Studies of a larger dataset that include assessment of the MIN status of tumors, for example by staining for the *hMLH1 *gene product, [41], will be needed to pursue this interesting correlation in depth.

Our study also revealed that stromal fascin staining was elevated in 47% of colorectal adenocarcinomas. This staining was independent of whether the adenocarcinoma itself was fascin-positive or -negative and tended to be strongest in the stroma adjacent to the invading front of the tumor. We postulate that increased stromal fascin represents an aspect of the host/tumor interaction. Many properties of the stroma, including extracellular matrix composition, density of immune and fibroblastic cells, angiogenesis and the production of angiogenic and chemoattractant factors, are known to be regulated as a result of crosstalk between the tumor and its surrounding host tissue [6]. The increased stromal fascin staining could reflect either a higher density of fascin-positive cells, most likely fibroblasts, or an increased fascin content per cell. From the Ki67 staining studies, it was clear that elevated stromal cell fascin did not correlate with zones of increased stromal cell proliferation. Further studies are needed to examine whether elevated stromal fascin staining is specific to colorectal adenocarcinomas or is also associated with carcinomas in other tissues.

Previous studies have suggested a possible relationship between fascin expression and increased proliferation of epithelial cells. Over-expression of fascin in colonic epithelial or its depletion in esophageal carcinoma cells correlated, respectively, with increased or decreased cell proliferation in culture [26,42]. The most strongly staining fascin-positive non-small cell lung carcinomas tended to be the most highly proliferative tumors, but cells high for Ki67 tended to be low for fascin [19]. However, the reverse trend was documented in gastric tumors, which had a higher Ki67 index in fascin-positive areas compared to fascin-negative areas [21]. Our analysis of colorectal adenomas and adenocarcinomas established that, in the adenomas, fascin-positive cells and crypts were clearly less proliferative than fascin-negative crypts (Fig. 1m, 1n). In the adenocarcinomas, distinctions between the fascin and Ki67 staining patterns were less clear-cut, but the overall trend was for fascin-positive cells to be low or negative for Ki67 and *vice versa*. Overall, our results do not support the hypothesis that up-regulation of fascin correlates positively with cell proliferation. There is accumulating evidence that migration and proliferation are exclusive behaviors for carcinoma cells and that migrating cells tend to be concentrated at the invading edge of tumors [43,44]. Thus, tumors containing a large fraction of fascin-positive cells might have a high potential for invasive behavior. Indeed, in breast carcinomas, fascin has been identified as a component of a gene signature that correlates clinically with tumor metastasis to the lung [25]. This concept would be in agreement with the correlation of fascin expression with an aggressive subset of advanced colon adenocarcinomas, as we have established from our TMA study.

## Conclusion

We found that the actin-bundling protein fascin is expressed in a subset of both adenomas and colorectal adenocarcinomas. Strong and widespread expression of fascin is detected in 16% of adenomas and between 17% to 26% of adenocarcinomas. In 47% of colorectal tumors, fascin was elevated in the surrounding stroma independent of fascin expression in the tumor. In adenomas, fascin and Ki67 tended to be inversely correlated at the cellular level; this trend was less clear in adenocarcinomas. In advanced tumors, strong tumor fascin staining correlated significantly with poor survival. Fascin has known roles in cell morphology and migration and may represent a potential novel marker or therapeutic target for the identification and treatment of patients with aggressive forms of colorectal adenocarcinoma.

## Abbreviations

CIN, chromosomal instability; CRC, colorectal carcinoma; ECM, extracellular matrix; FAP, familial adenomatous polyposis; MIN (MSI), microsatellite instability; TMA, Tissue microarray; TNM, tumor node metastasis stage classification.

## Competing interests

The author(s) declare that they have no competing interests.

## Authors' contributions

YH carried out scoring of sections, conducted the statistical analyses, prepared figures and tables and helped draft the manuscript. MS built the TMA, collected tumor specimens, organized the stainings, scored sections and contributed to the design of the study and the drafting of the manuscript. ICL contributed tumor specimens and in the setupof the CCF IRB-approved database. ALM participated in the preparation of the TMA and setup of the CCF IRB-approved database. GC participated in the preparation of the TMA and setup of the CCF IRB-approved database and contributed to the coordination of the study. JCA designed the study, participated in data analysis and drafted the manuscript. All authors read and approved the final manuscript.

## Pre-publication history

The pre-publication history for this paper can be accessed here:



## Figures and Tables

**Table 1:** Fascin positivity in adenomas and adenocarcinomas

	Fascin-positive cells			
	
	0	1–10%	10–50%	50%+
FAP (n = 18)				
n	5	12	1	0
%	28	67	6	0

Sporadic adenoma (n = 89)				
n	19	54	12	4
%	21	61	14	5

Adenocarcinoma (n = 35)				
n	9	17	3	6
%	26	49	9	17

Adenocarcinoma (TMA) (n = 131)				
n	93	16	16	6
%	71	12	12	5

**Table 2:** Clinicopathological characteristics of patients in the TMA dataset

Variables	n = 158	n = 131
Age		
<65 yrs	63	51
≥65 yrs	95	80

Gender		
Male	92	79
Female	66	52

Stage		
I/II	83	69
III/IV	75	62

Lymph node metastasis		
Negative	93	78
Positive	65	53

Location		
Proximal	43	37
Distal	115	94

**Table 3:** Relationship between fascin immunoreactivity and clinicopathologic characteristics

Variables	Fascin			
		
	0/1+	2+	3+	
	n = 113	n = 12	n = 6	*p*
Age				0.393
<65 yrs	44	6	1	
≥65 yrs	69	6	5	

Gender				0.885
Male	69	7	3	
Female	44	5	3	

Stage				0.971
I/II	60	6	3	
III/IV	53	6	3	

Lymph node metastasis				0.881
Negative	68	7	3	
Positive	45	5	3	

Location				0.009*
Proximal	29	3	5	
Distal	84	9	1	

**Table 4:** Multivariant analysis of the TMA study set

a. Cox's proportional hazard analysis in stage I to IV patients		
		
Variables	Risk ratio (95% confidence interval)	*p*
Age (≥65y)	1.618(0.944–2.774)	0.080
Gender (male)	0.615(0.365–1.036)	0.068
Lymph node metastasis (N1,2)	3.952(2.276–6.861)	<0.001*
Distant metastasis (M1)	1.720(0.938–3.155)	0.797
Tumor location (distal)	1.039(0.593–1.820)	0.894
Fascin (high)	1.184(0.905–3.676)	0.925
		
b. Cox's proportional hazard analysis in stage III and IV patients		
		
Age (≥65y)	1.259(0.683–2.318)	0.460
Gender (male)	0.603(0.322–1.129)	0.114
Lymph node metastasis (N1,2)	0.969(0.336–2.791)	0.934
Distant metastasis (M1)	1.245(0.539–2.628)	0.566
Tumor location (distal)	1.464(0.776–2.765)	0.240
Fascin (high)	2.301(1.045–5.068)	0.039*

**Table 5:** Relationship between Ki67 immunoreactivity and clinicopathologic characteristics

Variables	Ki67			
		
	1+	2+	3+	
	n = 64	n = 51	n = 16	*p*
Age				0.314
<65 yrs	23	19	9	
≥65 yrs	41	32	7	

Gender				0.354
Male	39	28	12	
Female	25	23	4	

Stage				0.048*
I/II	39	20	10	
III/IV	25	31	6	

Lymph node metastasis				0.467
negative	41	27	10	
positive	23	24	6	

Location				0.264
Proximal	15	15	7	
Distal	49	36	9	
