# Prognostic significance of fascin expression in advanced colorectal cancer: an immunohistochemical study of colorectal adenomas and adenocarcinomas

**DOI:** 10.1186/1471-2407-11-488

**Published:** 2011-11-21

**Authors:** Yosuke Hashimoto, Marek Skacel, Ian C Lavery, Abir L Mukherjee, Graham Casey, Josephine C Adams

**Affiliations:** 1 Department of Cell Biology, Lerner Research Institute, The Cleveland Clinic, 9500 Euclid Avenue, Cleveland, OH 44195, USA; 2 Department of Anatomic Pathology, The Cleveland Clinic, 9500 Euclid Avenue, Cleveland, OH 44195, USA; 3 Department of Colorectal Surgery, The Cleveland Clinic, 9500 Euclid Avenue, Cleveland, OH 44195, USA; 4 Department of Cancer Biology, Lerner Research Institute, The Cleveland Clinic, 9500 Euclid Avenue, Cleveland, OH 44195, USA; 5 Department of Molecular Medicine, Cleveland Clinic Lerner College of Medicine at Case Western Reserve University, The Cleveland Clinic, 9500 Euclid Avenue, Cleveland, OH 44195, USA

## 

Correction [1]: It was recently brought to our attention that, although the statistical analyses in Table four had passed the original peer-review of this paper, the risk ratio and value of the lower 95% confidence interval for some of the variables in Table four were not consistent with the *p *values. We have repeated the multi-variant statistical analysis of the original data with StatView software. This analysis produced different values for the risk ratios and 95% confidence intervals for variables other than age. The corrected Table four is presented below (table 1). The *p *values are unchanged, thus the original conclusions of the article are not altered.

**Table 1:** Corrected table four

Table 4a. Cox's proportional hazard analysis in stage I-IV patients		
**Variables**	**Risk ratio (95% confidence Interval)**	***p***

Age (≥ 65 yrs)	1.618 (0/944-2.774)	0.080
Gender (male)	1.626 (0.965-2.740)	0.068
Lymph node metastasis (N1, 2)	0.253 (0.146-0.439)	< 0.001*
Distant metastasis (M1)	0.581 (0.317-1.066)	0.797
Tumour location (distal)	0.963 (0.550-1.686)	0.894
Fascin (high)	0.548 (0.272-1.104)	0.925

Table 4b. Cox's proportional hazard analysis in stage III/IV patients		

Variable	Risk ratio (95% confidence Interval)	*p*

Age (> 65 yrs)	1.259 (0.683-2.318)	0.460
Gender (male)	1.659 (0.886-3.108)	0.114
Lymph node metastasis (N1, 2)	1.032 (0.358-2.972)	0.934
Distant metastasis (M1)	0.803 (0.381-1.697)	0.566
Tumour location (distal)	0.683 (0.362-1.289)	0.240
Fascin (high)	0.435 (0.197-0.957)	0.039*

## Pre-publication history

The pre-publication history for this paper can be accessed here:

http://www.biomedcentral.com/1471-2407/11/488/prepub
